# Median effective dosage of midazolam oral solution for preschool children in preoperative sedation

**DOI:** 10.1002/ibra.12170

**Published:** 2024-07-15

**Authors:** Rui Jiang, De‐Chuan Wang, Nan Zhao, Ke An, Yi‐Nan Zhang, Zhao‐Qiong Zhu

**Affiliations:** ^1^ Department of Anesthesiology Affiliated Hospital of Zunyi Medical University Zunyi China; ^2^ Department of Anesthesiology, Hospital of Stomatology Zunyi Medical University Zunyi China

**Keywords:** median effective dosage, midazolam oral solution, preoperative sedation, preschool children

## Abstract

This study aimed to detect median effective dosage (ED_50_) of midazolam oral solution (MOS) for preschool children in preoperative sedation. Thirty children (3–6 years old, with a body mass index (BMI) of 18–28 kg/m^2^, American Society of Anesthesiologists status (ASA) I‐II) scheduled for the hidden penis correction surgery under general anesthesia were selected. The effective dosage of MOS for preschool children in preoperative sedation was measured by sequential method. The initial dose was set at 0.5 mg/kg, with a concentration gradient of 0.1 mg/kg. Sedation was deemed successful if the patients’ Ramsay sedation scores were ≥4 and Frankl treatment compliance rating scale was ≥3, without any grade III or higher adverse events during anesthesia. If these criteria were met, the dosage for the next patient was reduced by one gradient based on the last patient's dosage, otherwise, the dosage for the next patient was increased by one gradient. This process was repeated until the 7th inflection point from unsuccessful to successful sedation was reached, at which point the trial was terminated. Probit regression analysis was used to calculate the ED_50_, 95% effective doses (ED_95_) and 95% confidence interval (CI) of MOS. Adverse reactions such as bradycardia, nausea, vomiting, and blurred vision were recorded during sedation. This study revealed that the ED_50_ and ED_95_ of MOS for preschool children preoperative sedation are 0.627 mg/kg (95% CI 0.582–0.669 mg/kg) and 0.795 mg/kg (95% CI 0.712–1.211 mg/kg), respectively, providing a reference for the administration of MOS in this population.

## Graphical abstract

This study aimed to detect median effective dosage (ED_50_) of midazolam oral solution (MOS) for preschool children in preoperative sedation. Thirty children who were scheduled for elective of the hidden penis correction surgery under general anesthesia in the pediatric surgery department were selected. The degree of anxiety before sedation was determined by Modification of the Yale preoperative anxiety scale. The Ramsay sedation scores and Frankl treatment compliance rating scale were measured to evaluate the degree of sedation after taking MOS. Obtained results show that MOS has a good sedative effect and can be safely used for preoperative sedation in preschool children.

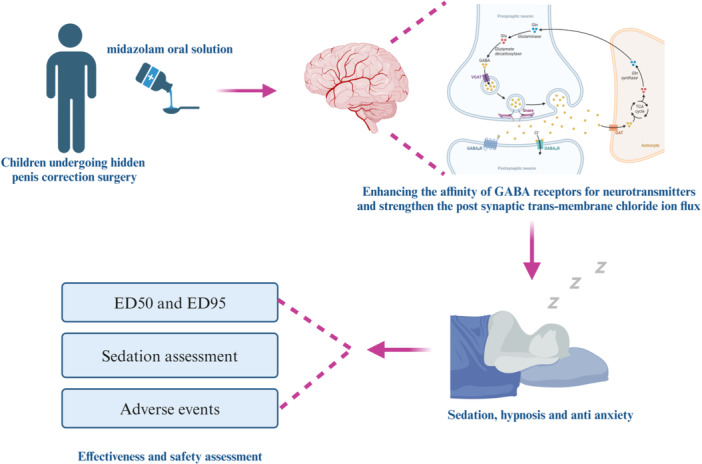

## 1 INTRODUCTION

Most preschool children showed negative emotions including nervousness, anxiety, and even fear when they encountered long‐time preoperative waiting, forceful separation from parents, and mandatory opening veins. Continuous cry and forceful anesthesia induction would trigger a range of airway complications, affect postoperative recovery, and even hamper physical and mental development, thus causing psychological and personality disorders in adulthood.^1^ Hence, appropriate preoperative sedation is vital for preschool children. How to make patients feel physiologically and psychologically pleasurable and comfortable during the perioperative period is a top concern for anesthesiologists.^2^ Midazolam is a benzodiazepine drug with anti‐anxiety, sedative, hypnotic, and anterograde amnestic effects. It helps patients remain alert and supports cognitive and psychological activity in the limbic system by acting on the ascending reticular tract of the midbrain and has anti‐anxiety effects. It has been widely applied in manual surgery and pre‐diagnostic sleep induction.^3^ Compared to other administration routes, midazolam oral solution (MOS) is a novel pediatric‐specific medication, the first of its kind in China, to exert preoperative sedative effects on children. It can make children less fearful of drug injection and intranasal administration and effectively alleviate preoperative anxiety in children, resulting in high satisfaction among both children and parents. MOS is mainly metabolized by cytochromes in the liver and intestine and is ultimately excreted by urine. The main adverse reactions include nausea and vomiting. However, there are few reports on studies of MOS applied to preschool children in China and the most effective dosage among this population has yet to be determined. Therefore, the present study aims at detecting median effective dosage (ED_50_) of MOS for preschool children in preoperative sedation to offer reference for clinical practices.

## 2 MATERIALS AND METHODS

### 2.1 General information

The present study was approved by the Ethics Committee of Affiliated Hospital of Zunyi Medical University (KLL‐2022‐623) and registered at the Chinese Clinical Trial Registry (registration number: ChiCTR2200062680). Informed consent was obtained from custodians of the patients. Thirty children (3–6 years old, with a body mass index (BMI) of 18–28 kg/m^2^, American Society of Anesthesiologists status (ASA) I‐II), who were scheduled for the hidden penis correction surgery under general anesthesia in the pediatric surgery department of Affiliated Hospital of Zunyi Medical University from August 2022 to November 2022, were selected. Patients with following conditions were excluded: (1) taking sedative or hypnotic drugs within recent 2 weeks; (2) having upper respiratory tract infection within recent 2 weeks; (3) severe cardiovascular diseases and mental system disease before the operation; (4) with custodians refusing to continue the trial.

### 2.2 Anesthesia

The patients were required to stop taking any food 8 h (h) before the operation and drinking water 2 h before the operation. On the day scheduled for the operation, anesthesiologists performed another anesthetic evaluation. Then, cardiac monitoring was conducted on the patients to monitor signs of life. Preoperative anxiety was evaluated based on Table 1. Subsequently, MOS (medicines vocabularies: Midazolam Oral Solution; manufacturer: Yichang humanwell Pharmaceutical Co., Ltd; batch number: 2L908031) was administered using sequential experimental method for preoperative sedation. Sedation assessment was performed at an interval of 30 min (min) after taking MOS. Specific evaluation content could be seen in the part 2.3 Sequential method. During sedation, ephedrine was administered when systolic blood pressure (SBP) was lower than 30% of basic values. Atropine was administrated when heart rate (HR) was less than 50 time/min. It is necessary to tilt back the patients' heads, raise their low jaws and let them absorb oxygen when oxygen saturation (SPO_2_) was over 90%. All the children involved in the study were followed up on the second day after operation.

**Table 1:** Modification of the Yale preoperative anxiety scale (m‐YPAS).

Section	Score	Observation and description
**Motion**	1	1. Looking around curiously, playing toys, reading, or doing activities appropriate to their age; Looking for toys or parents at waiting zones or treatment zones, or approaching to operation facilities;
	2	2. Never caring what was happening, eyes stared downwards, fiddling with fingers, or sucking thumbs or any other objects at hand; closely staying with their parents when waiting, or showing hyperactivity when playing;
	3	3. Unable to concentrate, putting down toys to look for parents; walking around without a specific purpose; walking or playing restlessly, moving randomly on operation beds, twisting their bodies, trying to take off face‐masks or unwilling to leave their parents;
	4	4. Trying to escape, struggling with limbs or twisting their bodies wildly; running without a purpose in waiting zones, never caring toys, unable to leave their parents, trying to grasp their parents.
**Utterance**	1	1. Reading, keeping raising questions and comments, talking to themselves, laughing, answering to questions quickly, being peaceful, or no inappropriate to social communication because of being too young or too concentrate to respond;
	2	2. Responding to adults in low voice, murmuring or just nodding;
	3	3. Remaining silent, no response to questions;
	4	4. Sobbing, groaning, muttering, crying quietly;
	5	5. Crying loudly or shouting out “No”;
	6	6. Keep crying and shouting loudly (can be head even with face‐masks on).
**Emotions**	1	1. Showing obvious joy, smiling, concentrating on playing;
	2	2. Expressionless;
	3	3. Too anxious to be afraid, feeling sad and uneasy, or eyes filled with tears;
	4	4. Feeling sorrowful, crying, remaining extremely uneasy, keeping eyes opening as widely as possible.
**Obvious alarming signs**	1	1. Alarming, occasionally looking around, caring or observing what anesthesiologists were doing (relaxation was possible);
	2	2. Wordless, sitting silently and alone, may suck their thumbs or bury their face in adults’ arms;
	3	3. Highly alarming, rapidly looking around, may be frightened by voice around, opening eyes wide;
	4	4. Sobbing nervously, or crying while push away others, turning around to escape.
**Reliance on parents**	1	1. Busy with playing, sitting at ease, or doing activities appropriate to their age without parents; able to cooperate and interact with their parents;
	2	2. Reaching out to their parents, talking with their parents quietly, actively seeking for comfort, may rely on parents;
	3	3. Quietly watching their parents, seemingly observing their actions, not actively touching or seeking for comfort but would accept it when parents actively offered it, closely clinging to parents;
	4	4. Keeping a distance with their parents or actively leaving them, may push away parents or closely clinging to them, not allowing them to leave.

*Note*: 1‐4 or 1‐6 points are given based on the number of items in each section. Then the score is converted to a 100‐point scale. Specific conversion method: actual scores = (scores of each section/item numbers) × (100/section numbers). The sum of actual scores of each section equals total scores. Higher scores indicate severe anxiety.

### 2.3 Sequential method

**Figure 1:**
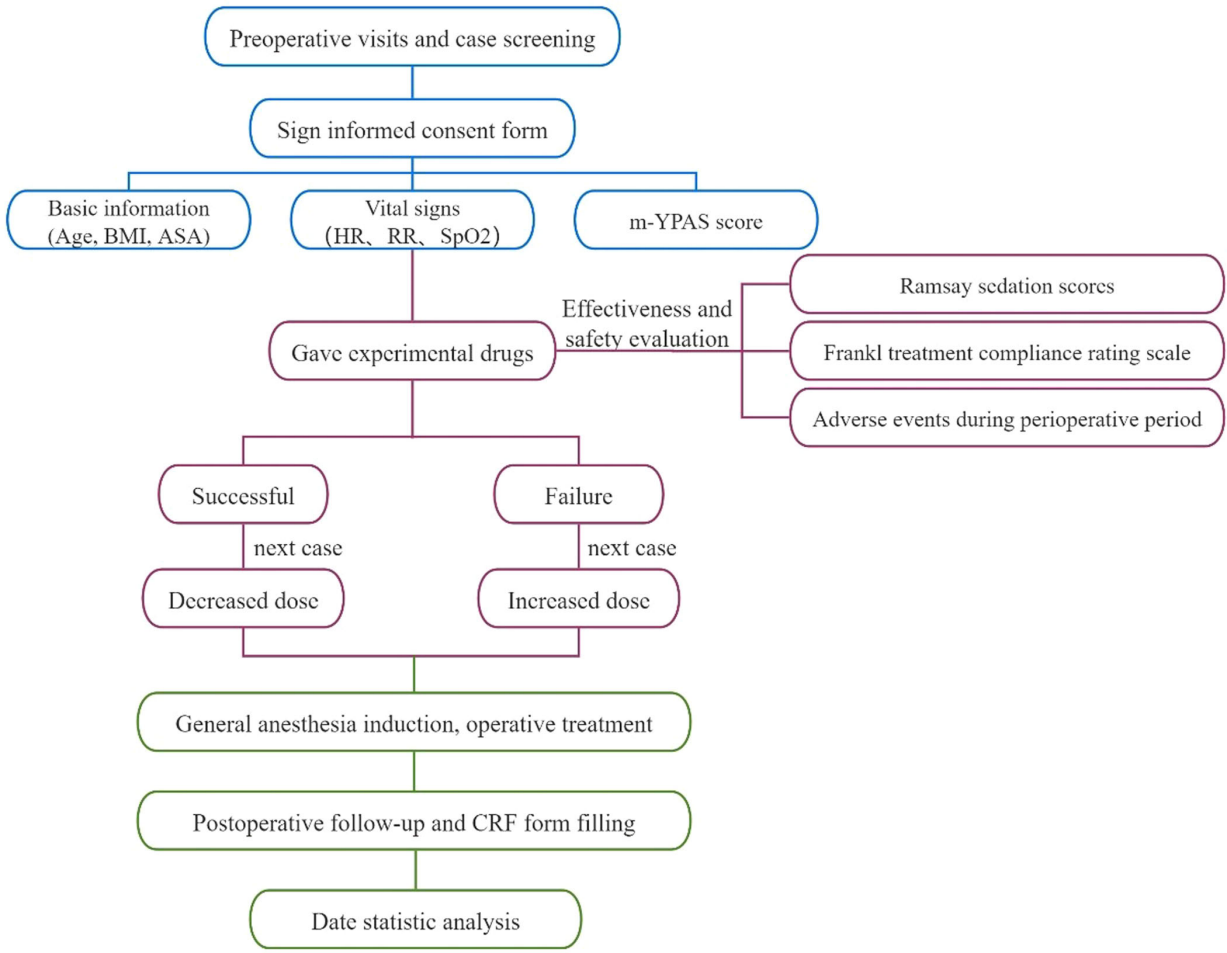
The test flowchart. If Ramsay sedation scores were ≥4, Frankl treatment compliance rating scale was ≥3, without level III or above adverse events during the anesthesia, sedation was successful; if not, the sedation is judged to be failure. ASA, American Association of Anesthesiologists; BMI, body mass index; HR, heart rate; RR, respiratory rate; SPO_2_, pulse oxygen saturation; m‐YPAS, modification of the Yale preoperative anxiety scale. [Color figure can be viewed at wileyonlinelibrary.com]

According to the reference^4^ and the present study, the initial dose was 0.5 mg/kg and the concentration gradient was 0.1 mg/kg. If patients' Ramsay sedation scores were ≥4 (Table 2) and Frankl treatment compliance rating scale was ≥3 (Table 3) without level III or above adverse events (Table 4) during the anesthesia, sedation was successful. Dosage for the next patient was reduced by a gradient based on the dosage for the last patient. Failure to achieve any of the above indicators was considered an unsuccessful sedation. Dosage for the next patient was increased by a gradient based on the dosage for the last patient. The above procedures were repeated until the 7th inflection point from unsuccessful sedation to successful sedation. Then, the trial was terminated. The test flowchart is presented in Figure 1.

**Table 2:** Ramsay sedation scores.

Score	Description
1	Anxious and restless
2	Cooperative, self‐restrained and silent
3	Responding to instructions
4	Drowsy and swift reaction to light blow to eyebrows or loud auditory stimulus
5	Drowsy and slow reaction to light blow to eyebrows or loud auditory stimulus
6	Drowsy and no reaction

**Table 3:** Frankl treatment compliance rating scale.

Rating	Description	Score
Complete refusal	Refusing to receive treatment, crying hard, feeling extremely terrified, utterance or expressions showing obvious reluctance to receive treatment	1
Relative refusal	Receiving treatment but unwilling to do so	2
Relative cooperation	Able to receive treatment, feeling cautious	3
Complete cooperation	Actively receiving treatment, harmony with doctors	4

**Table 4:** Adverse events during perioperative period.

Level	Description
I	Mild, no or minor clinical symptoms, unnecessary to take treatment
II	Middle, taking minor, local or noninvasive treatment, limitation on daily activities or using tools appropriate to age
III	Severe, not threatening life temporarily; hospitalization or prolonging hospital‐stays, paralysis, limitation on self‐care in daily life
IV	Threatening life, necessary to receive urgent treatment
V	Death

### 2.4 Observation indicators

The following information and data were recorded: patients' anxiety scores before administration, Ramsay sedation scores, Frankl treatment compliance rating scale, and adverse events during perioperative period.

### 2.5 Statistical analyses

The data were analyzed using SPSS 23.0. Measures conforming to normal distribution were expressed as mean ± standard deviation ($\bar{\chi }\pm s$). Count data were expressed as frequencies and t‐tests were used for comparisons between groups. Probit regression analysis was used to calculate the ED_50_, 95% effective doses (ED_95_) and 95% confidence interval (CI) of MOS. *p* ＜ 0.05 was considered statistical significance.

## 3 RESULTS

The present study included 30 cases as 14 cases from the successful group while 16 cases from the failure group. There was no statistical significance in age, height, weight, preoperative anxiety scores, and vital signs in both groups (*p *＞ 0.05) (Tables 5 and 6).

**Table 5:** General comparison between two groups ($\bar{\chi }\pm s$).

Group	Case	Age	Height	Weight	m‐YPAS score
Successful group	14	4.14 ± 0.95	106.36 ± 8.09	17.92 ± 3.12	42.98 ± 10.58
Failure group	16	4.38 ± 1.15	109.82 ± 7.62	18.32 ± 3.78	39.01 ± 11.64

Abbreviation: m‐YPAS, Modification of the Yale preoperative anxiety scale.

**Table 6:** vital signs comparison between two groups ($\bar{\chi }\pm s$).

Group	Case	HR	RR	SPO_2_
Successful group	14	97.86 ± 9.67	20.14 ± 1.46	98.29 ± 1.33
Failure group	16	95.56 ± 5.72	21.75 ± 1.8	98.19 ± 1.72

Abbreviations: HR, heart rate; RR, respiratory rate; SPO_2_, oxygen saturation.

**Figure 2:**
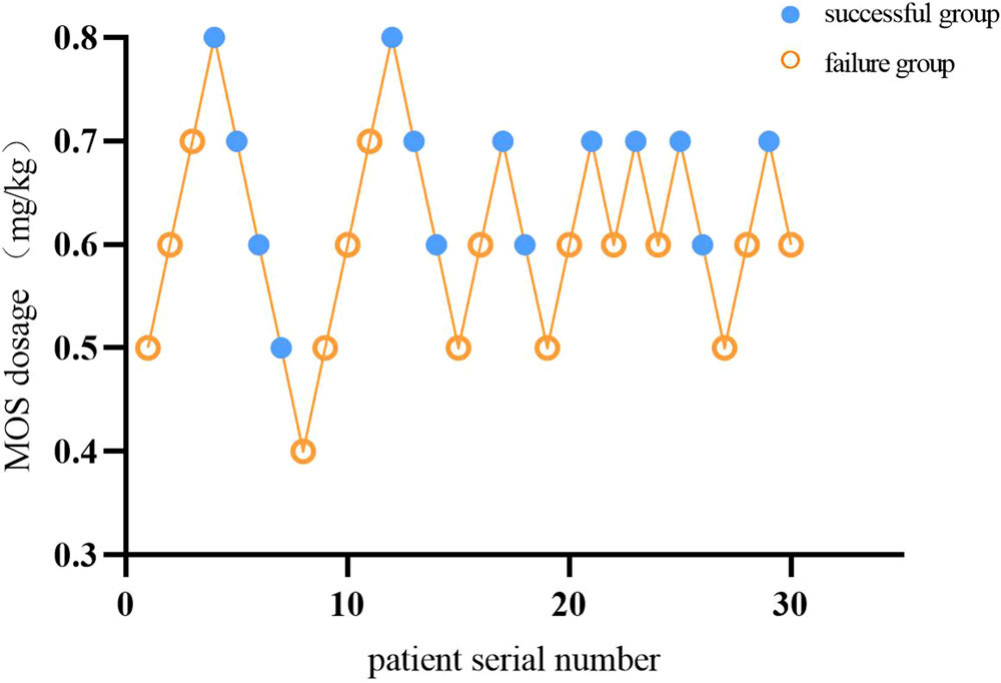
Sequence chart of MOS for preschool children in preoperative sedation. MOS, midazolam oral solution. [Color figure can be viewed at wileyonlinelibrary.com]

**Figure 3:**
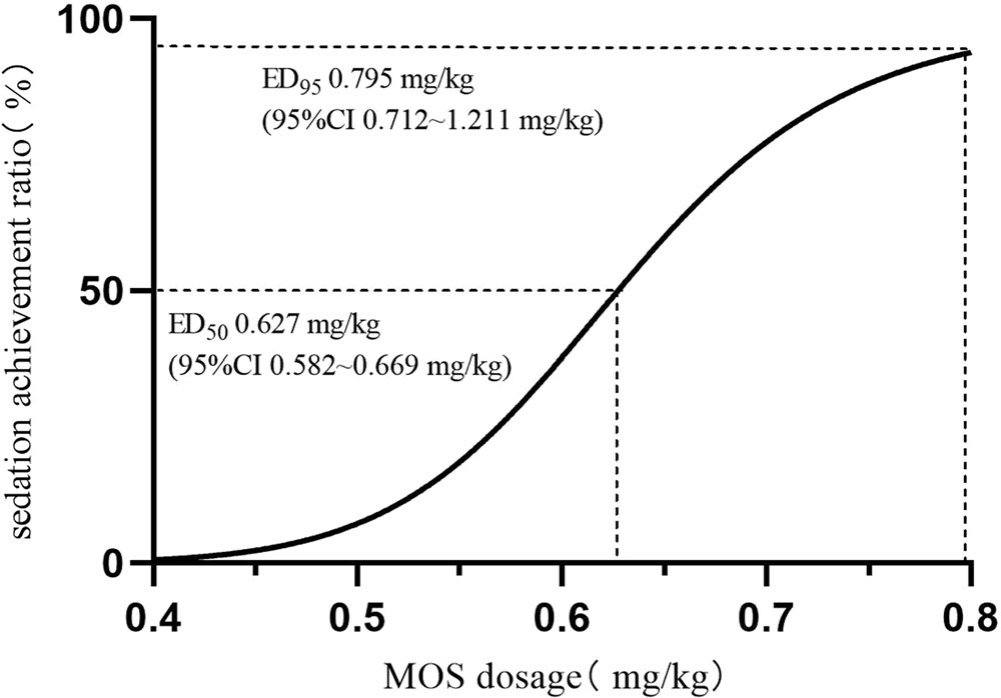
Dosage‐effect relationship chart of MOS for preschool children in preoperative sedation. CI, confidence interval; ED_50_, median effective dosage; ED_95_, 95% effective dosage; MOS, midazolam oral solution.

Importantly, we determined that the ED_50_ and ED_95_ of MOS for preschool children in preoperative sedation are 0.627 mg/kg (95% CI 0.582–0.669 mg/kg) and 0.795 mg/kg (95% CI 0.712–1.211 mg/kg), respectively (Figures 2 and 3).

No adverse reaction was found in the successful group. All the patients became sedative 30 min after administration. In the failure group, one patient began to cry 20 min after taking MOS. Another patient showed weakness of limbs but remained conscious 25 min after taking MOS. After re‐evaluation by the researchers, the above two children did not undergo special treatment. For children from the failure group, no additional experimental drugs were added. After the waiting time for surgery, anesthesia induction was performed according to the conventional procedure. The Ramsay sedative scores were all no more than 4 points in the failure group. No serious adverse reactions were found in the failure group during postoperative follow‐up.

## 4 DISCUSSION

Preschool period is vital for mental and personality development of children. Because of limitation on recognition, lack of self‐control and resistance against medical facilities and operation, children are more anxious and terrified than adult patients.^5^, ^6^ Forceful separation from parents, the closed environment of the operating room, fear of the opening vein procedure, and forceful anesthetic induction can exacerbate children's anxiety and fears. Hence, it would trigger many adverse reactions during anesthetic induction and affect postoperative recovery. What's worse, children's physical and psychological health and development would be hampered either in a short time or in a long time.^7^ As the society is progressing, anesthesiology shifts from securing patients during the perioperative period to offering more efficient and better medical services to patients throughout the treatment, thus making them feel physically and psychologically relieved and comfortable.^8^ Therefore, the major purpose of developing comfortable medicine lies in reducing children's anxiety and negative emotions because of separation from their parents with anesthetic techniques or medicine. Then, children are no longer terrified of check‐ups or treatment so that they are willing to cooperate in the following anesthetic procedure. For the first time, the present study detected and confirmed the effective dosage of MOS for preschool children in preoperative sedation and explore the security and efficacy of MOS. Of all 30 patients, one patient cried and another one showed weakness of limbs. Adverse events including illusion and diplopia did not occur. The result is similar to those of other studies.^9^ This study proved that oral administration, in spite of low bio‐availability, did not trigger adverse events with relatively large dosage than those in previous studies.^10^ Therefore, it confirmed that MOS was secure and effective for preschool children in preoperative sedation.

Studies have shown that preoperative sedative drugs effectively reduce children's anxiety, contribute to anesthetic induction, and reduce the incidence of postoperative agitation in children, thus protecting their psychological health.^11^ At present, midazolam is a common preoperative medication for children, with the advantages of fast onset, short duration, antagonistic effect, and safe and reliable sedative hypnotic and anti‐anxiety effects. The therapeutic effect and adverse reactions are achieved by activating the ascending reticular system γ‐ GABA receptors to inhibit the activity of the cortex and limbic system. However, midazolam tastes bitter and triggers hiccups. Inappropriate dosage would even lead to respiratory inhibition and cognitive disorders.^12^ In recent years, midazolam has been used with syrup in multiple clinical studies. Oral administration is safe and convenient, but plasma concentration and bio‐availability are hard to control, resulting in limited applications.^13^, ^14^ MOS is the first medication in China specifically indicated for sedation before and during preoperative, diagnostic, or therapeutic operations in children. MOS tastes slightly sweet and has higher bio‐availability, which has been well‐received by both children and their parents. Furthermore, oral administration is more comfortable than intramuscular injection, intravenous injection, nasal drop, and enema, thus providing a humane treatment environment for children. As a study shown, MOS and nasal administration have similar sedative effects and effectively relieve children's anxiety. However, MOS takes less time to take effect and is more acceptable for children.^15^ Currently, there is no report on studies of MOS applied in preschool children and its accurate usage in China. Therefore, in this study, the sequential experimental method was used to determine the dosage‐effect relationship and produced precise medication data to provide evidence and guidance for clinical medication.

Improved Dixon sequence method was applied in this study. The sequential test is commonly used to calculate the ED_50_ and ED_95_ of medicine. Clinically, anesthesiologists should not only pay attention to the ED_50_ of drugs but also apply ED_95_ to guide medication in more cases. Therefore, ED_50_ and ED_95_ were calculated in this study. Its advantages include saving samples and easy to conduct. The test result would accurately reflect the potency of medicine, so it is one of the classical methods to study dosage‐effect relationship.^16^ Based on the pre‐experimental results, the initial dose was 0.5 mg/kg and the concentration gradient was 0.1 mg/kg. The trial was terminated until the 7th inflection point was completed. Usually, 20–40 patients are included the sequential test.^17^ Therefore, 30 patients were eventually included in the study, which could fully reflect the reliability of the study. The ED_50_ and ED_95_ of MOS for preschool children preoperative sedation are 0.627 mg/kg (95% CI 0.582–0.669 mg/kg) and 0.795 mg/kg (95% CI 0.712–1.211 mg/kg) respectively. Cheng et al. found^18^ that MOS can be used for sedation, as well as hypnosis for children through a systematic review. They recommended a dosage of 0.5–1 mg/kg. Moreover, MOS is as effective as midazolam injection and dexmedetomidine and is superior to ketamine. However, the study targeted at 0–18‐year‐old patients and did not concentrate on age distribution. In contrast, the present study detected and confirmed the ED_50_ and ED_95_ of MOS for preschool children in preoperative sedation for the first time. The results of the study may be enlightening for the precise medication in preschool children.

Limitations of this study are listed as follows: (1) The inclusion criteria for patients and surgical methods are relatively homogeneous since the patients are limited to preschool children who underwent penile straightening. Further research is warranted to explore patients of different ages, genders, and surgical methods. (2) Sequence method that is used to study the dosage‐effect relationship is easy and requires fewer samples, but it is relative cursory. The ED_50_ and ED_95_ that are originated from this study could offer reference for clinical practice. However, further randomized controlled trials are warranted to investigate more effective medication and dosage.

In all, the ED_50_ and ED_95_ of MOS for preschool children preoperative sedation are 0.627 mg/kg (95% CI 0.582–0.669 mg/kg) and 0.795 mg/kg (95% CI 0.712–1.211 mg/kg), respectively. The sedative effects are precise, with effective preoperative sedation for children and no serious adverse events, providing preschool children with a basis for medication and comfort medical care.

## AUTHOR CONTRIBUTIONS

Rui Jiang, De‐Chuan Wang, and Ke An collected data. Nan Zhao wrote the original draft. Yi‐Nan Zhang edited the draft, and Zhao‐Qiong Zhu supervised the work. All authors have read and agreed to the published version of the manuscript.

## CONFLICT OF INTEREST STATEMENT

The authors declare no conflict of interest.

## ETHICS STATEMENT

The study was approved by the Ethics Committee of the Affiliated Hospital of Zunyi Medical University (approval number: KLL‐2022‐623) and registered at the Chinese Clinical Trial Registry (registration number: ChiCTR2200062680). Informed consent was obtained from custodians of the patients.

## Data Availability

The data supporting this study are available on request from the corresponding author.
